# Fabrication of Microbolometer Arrays Based on Polymorphous Silicon–Germanium

**DOI:** 10.3390/s20092716

**Published:** 2020-05-09

**Authors:** Ricardo Jimenez, Mario Moreno, Alfonso Torres, Alfredo Morales, Arturo Ponce, Daniel Ferrusca, Jose Rangel-Magdaleno, Jorge Castro-Ramos, Julio Hernandez-Perez, Eduardo Cano

**Affiliations:** 1 Electronics Group, National Institute of Astrophysics, Optics and Electronics, Puebla 72840, Mexico; rjzavala@inaoep.mx (R.J.); atorres@inaoep.mx (A.T.); alfredom@inaoep.mx (A.M.); aponce@inaoep.mx (A.P.); dferrus@inaoep.mx (D.F.); jrangel@inaoep.mx (J.R.-M.); jcastro@inaoep.mx (J.C.-R.); julio.hernandez@inaoep.mx (J.H.-P.); eduardo.canohe@my.uvm.edu.mx (E.C.); 2 Department of Physics and Astronomy, University of Texas at San Antonio, San Antonio, TX 78249, USA

**Keywords:** infrared, sensor, microbolometer, array, polymorphous, silicon, germanium, plasma-enhanced chemical vapor deposition

## Abstract

This work reports the development of arrays of infrared sensors (microbolometers) using a hydrogenated polymorphous silicon–germanium alloy (pm-Si_x_Ge_1-x_:H). Basically, polymorphous semiconductors consist of an amorphous semiconductor matrix with embedded nanocrystals of about 2–3 nm. The pm-Si_x_Ge_1-x_:H alloy studied has a high temperature coefficient of resistance (TCR) of 4.08%/K and conductivity of 1.5 × 10^−5^ S∙cm^−1^. Deposition of thermosensing film was made by plasma-enhanced chemical vapor deposition (PECVD) at 200 °C, while the area of the devices is 50 × 50 μm^2^ with a fill factor of 81%. Finally, an array of 19 × 20 microbolometers was packaged for electrical characterization. Voltage responsivity values were obtained in the range of 4 × 10^4^ V/W and detectivity around 2 × 10^7^ cm∙Hz^1/2^/W with a polarization current of 70 μA at a chopper frequency of 30 Hz. A minimum value of 2 × 10^−10^ W/Hz^1/2^ noise equivalent power was obtained at room temperature. In addition, it was found that all the tested devices responded to incident infrared radiation, proving that the structure and mechanical stability are excellent.

## 1. Introduction

Semiconductor materials with characteristics of low resistivity and high temperature coefficient of resistance (TCR) are important for detection of infrared radiation by uncooled microbolometers, which are widely used in the range of 8–12 μm, known as LWIR (long-wave infrared). A microbolometer is a thermal detector where a resistive membrane absorbs infrared radiation, which increases its temperature. For semiconductor materials, the increase in temperature reduces the resistance of the membrane and the measurement of the change of resistance is the output of the sensor. A high TCR is desirable to increase the sensitivity of the device or maintaining it when it is desired to reduce the pixel area. On the other hand, a low resistivity makes it easier to read the pixels with the associated ROIC (readout integrated circuit). In addition, the resistivity of the material directly influences the 1/f noise and consequently the performance of the devices.

In relation to the electrical characteristics described above, several materials have been studied for their application as thermosensing films, including carbon nanotubes and other organic compounds with TCR values of about 2.1%–3.1%/K [1,2,3], poly-SiGe (0.68%–2%/K) [4], Y-Ba-Cu-O (~3.3%/K) [5], metal films Pt (~0.14%/K) [6], Ti (~0.35%/K) [7,8]; as well ZnO has shown high TCR (~10.4%/K) in thin films, but without reported experimental results on devices [9].

At the present time, vanadium oxide (VO_x_) (~2%/K) [10,11,12,13,14,15,16] and doped amorphous silicon (a-Si) with TCR values of 2%–5%/K [17,18,19,20,21,22] are the two main materials used as thermosensing films in commercial microbolometers. However, amorphous silicon, usually doped with boron, is replacing vanadium oxide in large commercial arrays. The reason is the full silicon complementary metal oxide semiconductor (CMOS) processes compatibility and the larger TCR of amorphous silicon. This means that large arrays can be fabricated in standard silicon CMOS facilities, and moreover, those arrays contain devices with better performance characteristics.

In this work, we study the implementation in microbolometer arrays of a hydrogenated polymorphous silicon-germanium alloy (pm-Si_x_Ge_1-x_:H), which is a material composed of an amorphous matrix with embedded nanocrystals in the range of 2–3 nm [23]. The presence of nanocrystals reduces the density of states, improving the transport properties and the stability of the material against radiation-induced degradation (Staebler–Wronski effect) [24]. This was confirmed with the results of aged polymorphous silicon (pm-Si:H) solar cells by light-soaking [25]. In pm-Si_x_Ge_1-x_:H, the TCR can be easily modified with the silicon/germanium ratio of the alloy, and as a consequence the 1/f noise is also modified [26,27]. This novel material is deposited by plasma-enhanced chemical vapor deposition (PECVD) at low temperature and pattern definition can be performed by dry-etching techniques, which are conventional steps in integrated circuits processing; moreover, their TCR values are larger than those obtained in doped amorphous silicon. As a result, pm-Si_x_Ge_1-x_:H is a material with full CMOS processes compatibility and can also be synthesized at low temperatures, making it ideal for microbolometer implementation.

In previous work, titanium (Ti) electrodes were on the top of the membrane [28,29], however, there were problems of stress mismatching between Ti and pm-Si_x_Ge_1-x_:H. Now we have decided to replace the coplanar electrodes with Ti studs; in addition, an array of microbolometers with pixel area of 50 × 50 μm^2^ was fabricated in order to study the stability of the structures and analyze their performance quality.

## 2. Materials and Methods

### 2.1. Preparation Method of the Thermosensing Film

Deposition of pm-Si_x_Ge_1-x_:H was made in a plasma-enhanced chemical vapor deposition (PECVD) reactor AMP-3300 from Applied Materials, Inc., Santa Clara, CA, USA, working at 110 kHz. A mixture of silane (SiH_4_) and germane (GeH_4_) gases were diluted in hydrogen with a ratio of 1:20. Nanocrystal formation is promoted when plasma conditions are close to powder regime [30], in that sense we set a relatively high pressure inside the chamber. The whole set of conditions is listed in Table 1.

### 2.2. Temperature Dependence of Conductivity

In order to measure dark conductivity as a function of temperature *σ(T)*, a pm-Si_x_Ge_1-x_:H film was deposited on top of coplanar titanium electrodes using Corning Glass 2947 as a substrate. The geometry of the electrodes was 300 nm thick, 9.5 mm long, and separated by 2 mm. *σ(T)* of the films was measured in the range of 300–400 K inside a vacuum thermostat VPF-100 from Janis Research Company, LLC, Woburn, M.A. USA, at 15 mTorr in steps of 10 K, while a ramp of 5 K/min was employed. At every point, the sample was allowed to thermalize for 3 min before each measurement. As humidity can play a major role in conductivity measurements [31], two heating ramps were carried out, one to desorb humidity and the second for data collection. The conductivity in pm-Si_x_Ge_1-x_:H shows a thermally activated Arrhenius behavior, which is related to activation energy (*E_a_*) through Equation (1), where *k* is the Boltzmann constant and *σ*_0_ the conductivity prefactor [19].

(1) $$\sigma (T)=\sigma_{0}\exp(-E_{a}/kT).$$

Solving for *E_a_* to fit the experimental data with a straight line, gives Equation (2):

(2) $$Ln(\sigma )=Ln(\sigma_{0})-E_{a}/kT.$$

The slope of the line gives *E_a_* and the *TCR* is finally calculated using Equation (3):

(3) $$TCR=(-E_{a}/kT^{2})\times 100.$$

Polymorphous silicon–germanium, like many semiconductors, exhibits a negative *TCR* (its resistance decreases with temperature), although for convenience the absolute value will be mentioned unless otherwise indicated.

**Figure 1:**
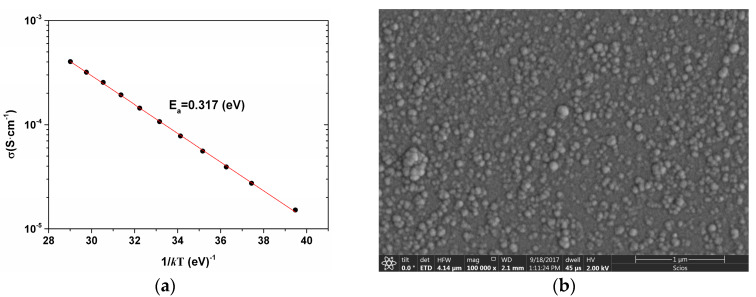
(**a**) Temperature dependence of conductivity for the pm-Si_x_Ge_1-x_:H film. (**b**) High-resolution field emission SEM (FE–SEM) image of the pm-Si_x_Ge_1-x_:H membrane surface.

Figure 1a shows an Arrhenius plot of conductivity as a function of temperature for the film deposited under the conditions given in Table 1. From that plot, the slope of the line gives us *E_a_* = 0.317 eV, which corresponds to a high *TCR* = 4.08%/K and a room temperature conductivity of *σ_RT_* = 1.5 × 10^−5^ S∙cm^−1^. Figure 1b shows a high-resolution field emission SEM (FE–SEM) image of the pm-Si_x_Ge_1-x_:H membrane surface, where several silicon–germanium nanoclusters are observed.

### 2.3. Microbolometer Fabrication

**Figure 2:**
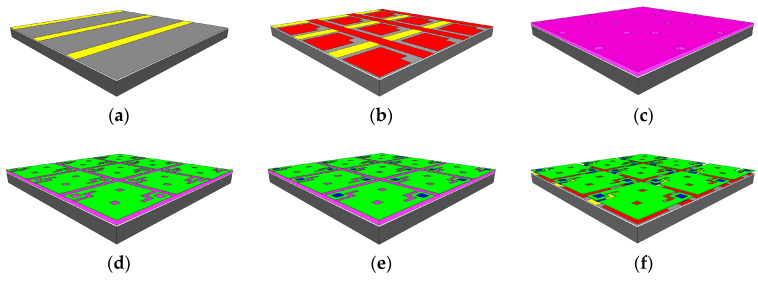
Fabrication process flow: (**a**) aluminum deposition and patterning (columns); (**b**) after deposition of the double layer of dielectric, a second layer deposition of aluminum (rows and mirror) followed; (**c**) polyimide deposition and opening of holes for contacts; (**d**) pm-Si_x_Ge_1-x_:H deposition and patterning; (**e**) deposition of titanium studs; (**f**) dry etching of the sacrificial polyimide layer.

The fabrication of the microbolometer array started with an oxidized silicon wafer, followed by the deposition of 600 nm of aluminum (Al) pad by e-beam evaporation, and defined by lithography and wet etching in Al-etch solution (80% H_3_PO_4_ + 5% HNO_3_ + 5% CH_3_COOH + 10% H_2_O at 40 °C, 10 min). Figure 2 shows the fabrication process.

Then, spin-on glass (SOG) 700B diluted in water at 50% was deposited on the wafer and cured at 250 °C for 2 h in air to obtain a layer with a refractive index *n* = 1.462 and a thickness of 180 nm that helps to planarize the surface. A refractive index close to that of thermal SiO_2_ assures a dense film with a low number of voids and defects. We avoided a thicker layer of SOG due to cracking and peeling issues by adding a second dielectric 120 nm-thick silicon nitride (SiN_x_) layer by PECVD. All film depositions by PECVD were performed in a system working at 110 kHz and 200 °C.

After that, a second Al metal layer with a thickness of 600 nm was evaporated and patterned by liftoff. The first Al layer defined the columns and the second layer defined the rows and the reflective mirror. Then, a 50 nm-thick SiN_x_ layer was deposited by PECVD to protect the aluminum pads from a later step of wet etching. A layer of polyimide (PI-2610) was spin-coated on the silicon wafer to form a 2.5 μm-thick gap, as part of a resonant quarter-wavelength cavity to improve absorption in the LWIR band. This Fabry–Perot resonant cavity was tuned at 10 μm wavelength by adjusting polyimide thickness d according to the relation *d* = λ/4*n* + Nλ/2*n*, where N = 0, 1, 2, ... and *n* is the refractive index of the vacuum (after polyimide removal). Polyimide was soft-backed in a nitrogen environment with a ramp of 5 °C/min and finally cured at 250 °C for 2 h in fresh air to avoid the formation of bubbles.

A hard Al mask, 150 nm thick, was evaporated and patterned by wet etching; the polyimide was then patterned with oxygen plasma by reactive ion etching (RIE) using an AME-8110 tool from Applied Materials, Inc., Santa Clara, CA, USA, in order to open contact windows. After that, Al-etch solution was employed to remove the aluminum hard mask. The following step consisted of patterning the SiN_x_ and SOG layers through the contact windows using CF_4_-based plasma with a micro-RIE series 800 reactor from Technics. Then, the thermosensing film 400 nm thick was deposited as described earlier (Section 2.1). In order to shape the membranes, the CF_4_ plasma was employed to pattern the pm-Si_x_Ge_1-x_:H film by the RIE technique.

After that, a 600 nm-thick Ti layer was evaporated and patterned by liftoff to create supporting studs. This structure thermally isolates the membrane from the substrate. Furthermore, Ti has a low thermal conductivity of 21.9 W/m⋅K and is a material commonly used in the Si–CMOS process. Finally, the silicon wafer was diced and the polyimide sacrificial layer was removed by oxygen plasma using a barrel asher L2101 from Branson/IPC, Hayward, CA, USA.

### 2.4. Experimental Setup

**Figure 3:**
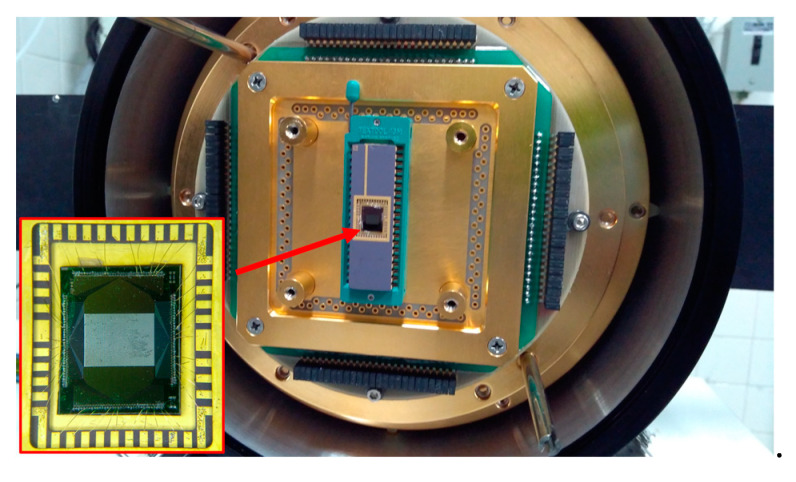
Array of microbolometers wire-bonded to a DIP-40 package and mounted inside the vacuum chamber.

To perform the characterization, a single die with an array of 19 × 20 elements was placed in a DIP-40 package from Kyocera Corporation, Kyoto, Japan and bonded with gold wires. Microbolometers were then mounted inside a vacuum chamber MTD-150 from Lake Shore Cryotronics, Inc., Westerville, OH, USA (Figure 3). It was evacuated to 20 mTorr and measurements were carried out at 300 K. For voltage responsivity (ℜ_*v*_) measurements, the array was illuminated with a cavity blackbody radiator using a silicon carbide (SiC) rod from Kanthal Globar as a broadband infrared source. Radiation comes out through a one inch aperture, which was then modulated at 30 Hz with a chopper wheel SR540 from Stanford Research Systems, Sunnyvale, OH, USA.

**Figure 4:**
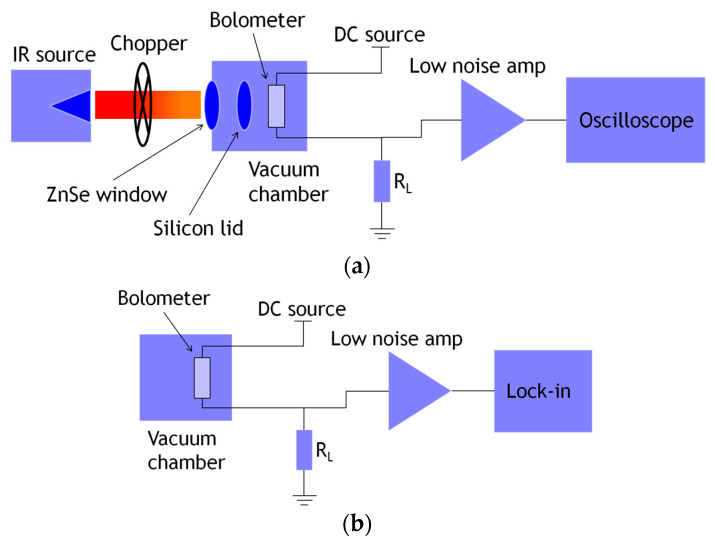
Experimental setup for (**a**) responsivity and (**b**) noise measurement.

Modulated radiation was then passed through a flat window of zinc selenide (ZnSe) with transmittance of 70% in the range of 0.6–20 μm, followed by placement of a 260 μm-thick silicon lid in front of the package. The incident radiation power was measured with an Oriel 71968 thermopile. A DC current of 70 μA was set across each microbolometer through a metal load resistor of 15 kΩ by using a SourceMeter-2400 from Keithley. The resistor value was very close to that of most measured bolometers. Voltage output was routed to a low noise amplifier LMC6482, from Texas Instruments, Inc., Dallas, TX, USA, configured as voltage follower and powered with batteries. Then, the output was connected to an oscilloscope; Figure 4a shows the setup employed. Noise measurements were performed in dark conditions at 300 K, and as before, a DC current of 70 μA was set. In addition, the vacuum inside the chamber was kept at 20 mTorr. The voltage signal was routed to a lock-in amplifier SR530 from Stanford Research Systems, as shown in Figure 4b. A reference signal of 30 Hz was provided from a signal generator, while measurements were performed with a bandwidth (Δ*f*) of 1 Hz.

## 3. Results

**Figure 5:**
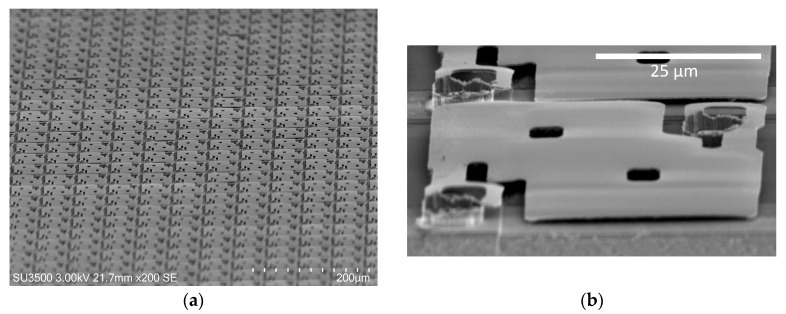
(**a**) SEM image of a part of the microbolometer array. (**b**) SEM image of one 50 × 50 µm^2^ microbolometer.

A scanning electron microscope (SEM) picture of a section of the microbolometer array is shown in Figure 5a, while Figure 5b shows an image of one 50 × 50 µm^2^ microbolometer. A close inspection shows a slight buckling of the membrane due to residual stress in the pm-Si_x_Ge_1-x_:H; however, it is also observed that the microbolometers in the array are in structurally good condition.

Voltage responsivity (ℜ_*v*_) was experimentally calculated in each microbolometer using Equation (4), where Δ*V* was the voltage output in response to the infrared signal *P_in_* irradiated to the microbolometer, calculated as 4.84 × 10^−7^ W from measurements of the thermopile and the device’s area. A maximum ℜ_*v*_ of 4 × 10^4^ V/W at 30 Hz chopper frequency was found.

(4) $${ℜ}_{V}=\frac{\Delta V}{P_{in}}.$$

Noise in microbolometers was calculated using Equation (5), where *V_nBol_*_+*System*_ is the root mean square (rms) noise of the bolometer plus the rms noise of the system, while *V_nSystem_* is the rms noise of the setup without the contribution of the microbolometer. *V_nSystem_* was found to be 32.8 nV/Hz^1/2^, and the noise in microbolometers was in the range 2–35 μV/Hz^1/2^.

(5) $${\left(\bar{V_{nBol}}\right)}^{2}={\left(\bar{V_{nBol+System}}\right)}^{2}-{\left(\bar{V_{nSystem}}\right)}^{2}.$$

Specific detectivity (D*) was then calculated with Equation (6) using data from the responsivity and noise measurements, where *A* is the effective area of the microbolometer, which was 2.046 × 10^−5^ cm^2^, and Δ*f* is the bandwidth, equal to 1 Hz. Detectivity is a signal-to-noise ratio normalized to the area of the detector, thus it can be used to compare performance between different types of detectors.

(6) $$D*=\frac{{ℜ}_{V}\sqrt{A\Delta f}}{\bar{V_{nBol}}}.$$

**Figure 6:**
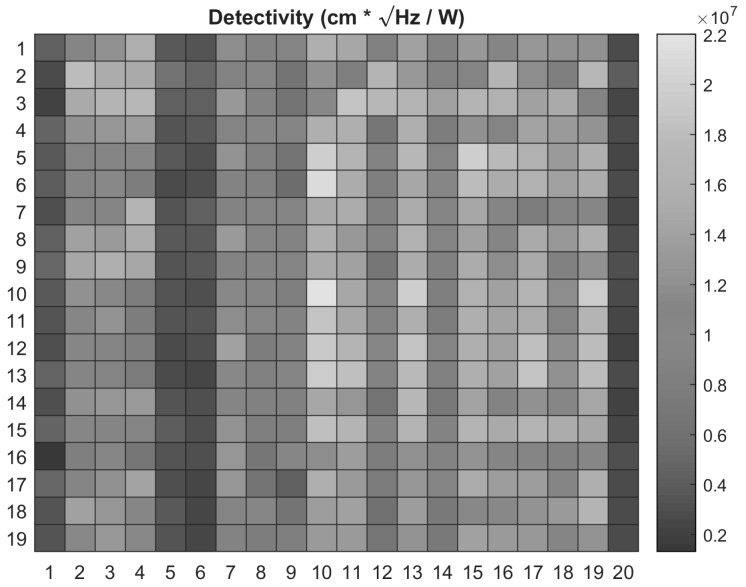
Detectivity map of the microbolometer array.

A detectivity map of the 380 measured devices is shown in Figure 6. A maximum D* was found to be 2 × 10^7^ cm∙Hz^1/2^/W, while the minimum was approximately 0.2 × 10^7^ cm∙Hz^1/2^/W. Furthermore, it was observed that all devices responded to infrared radiation, which is a consequence of the structural integrity of all the microbolometers in the array. This is of major importance since the percentage of damaged pixels in a commercial array is in the order of 1% [32]. The performance of the pm-Si_x_Ge_1-x_:H film implemented on a 19 × 20 microbolometer array serves as a proof-of-concept to assess its application on commercial devices. This material is an ideal candidate to reduce manufacturing cost as it can be readily integrated with CMOS back-end-of-the-line processes and ultimately to be applied in detectors of low-cost and high-volume markets [33].

In addition, we calculated the noise equivalent power (NEP) with Equation (7), which represents the amount of absorbed radiation power that gives a signal equal to the total rms noise. In terms of bolometer performance, the lower the *NEP* the better. Of all the analyzed devices, a minimum value of 2 × 10^−10^ W/Hz^1/2^ was found.

(7) $$NEP=\frac{\bar{V_{nBol}}}{{ℜ}_{V}}.$$

**Figure 7:**
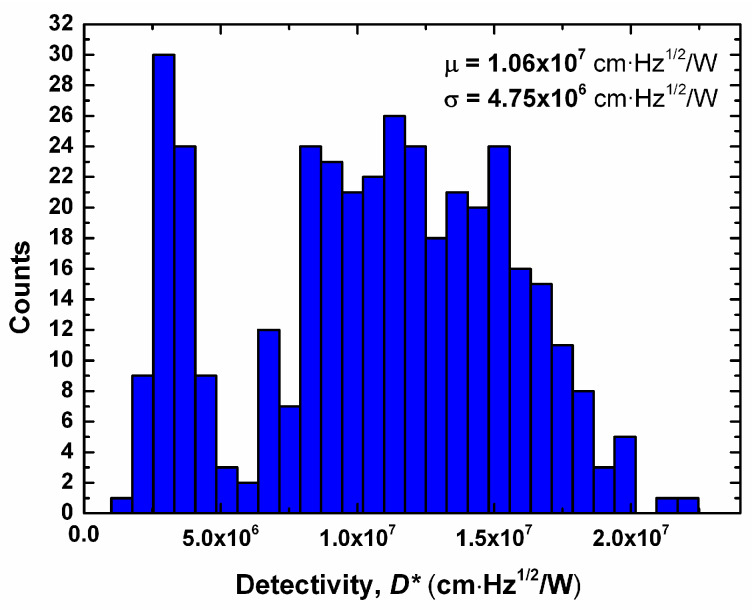
Histogram of the detectivity distribution in the microbolometer array.

Statistical analysis showed a mean detectivity (*µ*) of 1.063 × 10^7^ cm∙Hz^1/2^/W with a standard deviation (*σ*) of 4.75 × 10^6^ cm∙Hz^1/2^/W. The histogram of the detectivity values is shown in Figure 7, where Gaussian behavior was observed, which is commonly found in microbolometer arrays. However, a second Gaussian was also observed at lower D* values, which was a possible consequence of the stress presented in some microbolometers, causing a thermal flow to the substrate and thus a reduction of D*. Although a large standard deviation value is not desirable, it is important to stress that all devices showed a response under infrared radiation. The reduction of residual stress is a subject outside the scope of the present work; however, we achieved promising results with thermal treatments with temperatures as low as 200 °C.

In order to compare the performance of pm-Si_x_Ge_1-x_:H microbolometers with similar devices, Table 2 provides data for sensors having different thermosensing materials. First, we have a new approach to increase TCR by thermal annealing at 300 °C of a multilayer structure of VO_x_/ZnO. Here, detectivity in the order of 10^7^ cm∙Hz^1/2^/W is achieved; however, this material lacks CMOS compatibility. In the same way, the device with an active layer of VO_x_ requires thermal annealing at 300 °C to obtain a low noise level; this results in a D* value in the order of 10^7^ cm∙Hz^1/2^/W and low bolometer resistance (200 kΩ). Once again, this VO_x_ microbolometer cannot be integrated into a standard Si–CMOS process. The amorphous silicon (a-Si:H) active film device has an innovative pixel-level packaging, yet it has serious impedance problems due to the extensive number of manufacturing steps. This is reflected in a noise level that directly affects D* with a low value of 10^6^ cm∙Hz^1/2^/W. Nevertheless, this device boosts the current trend towards silicon-based microbolometers. Finally, a poly-SiGe device with a good balance between noise and TCR results in a high detectivity value of 10^9^ cm∙Hz^1/2^/W; despite being a material compatible with CMOS technology, it requires a crystallization process higher than 600 °C, which introduces serious thermal constraints.

Here we have presented the advantages of taking a material compatible with CMOS processes such as a-Si:H and improving it to obtain better performance. However, having a high TCR value is not enough, it is also necessary to optimize other properties such as noise and residual stress. In our case, it is probable that the low level of ℜ_*v*_ is the consequence of a thermal leak towards the substrate due to the bending of the membranes.

## 4. Conclusions

An array of 19 × 20 microbolometers made of pm-Si_x_Ge_1-x_:H was fabricated and tested. A thermosensing film with a TCR of 4.08%/K was employed and microbolometers showed a low resistance of about 15 kΩ. Noise was found to be in acceptable range of values (µV/Hz^1/2^), but responsivity was low, about 1 × 10^4^ V/W, and detrimental to detectivity, which achieved values in a range near 1 × 10^7^ cm∙Hz^1/2^/W at 30 Hz of chopper frequency. Finally, we found an exceptional operability of 100% of the pixels tested and the minimum NEP was 2 × 10^−10^ W/Hz^1/2^. Improvements in the performance of the devices for both detectivity and spatial uniformity are in progress.

## Figures and Tables

**Table 1:** Deposition parameters for the pm-Si_x_Ge_1-x_:H layer.

GeH_4_ (sccm)	SiH_4_ (sccm)	H_2_ (sccm)	Pressure (Torr)	Power (mW/cm^2^)	Temperature (°C)
30	20	1000	1.2	90	200

**Table 2:** Comparison of pm-Si_x_Ge_1-x_:H microbolometer performance with other devices at 30 Hz.

Material	pm-Si_x_Ge_1-x_:H This Work	VO_x_/ZnO [34]	a-Si:H [35,36]	VO_x_ [37]	Poly-SiGe [4]
**Size** (µm^2^)	50 × 50	50 × 50	35 × 35	25 × 25	50 × 50
**ℜ_*v*_** (V/W)	2 × 10^4^	2 × 10^4^	3.7 × 10^3^	2.44 × 10^5^	1.64 × 10^4^
**NEP** (W/Hz^1/2^)	2 × 10^−10^	1 × 10^−10^	7.7 × 10^−10^	1.4 × 10^−10^	6 × 10^−10^
D* (cm · Hz^1/2^/W)	2 × 10^7^	4 × 10^7^	4.5 × 10^6^	1.55 × 10^7^	2.26 × 10^9^
**TCR** (%/K)	4.08	3.12	2.27	2.6	0.68
**R** (Ω)	15 × 10^3^	−	200 × 10^9^	200 × 10^3^	10 × 10^3^
